# Malaria and Helminth Co-Infections in School and Preschool Children: A Cross-Sectional Study in Magu District, North-Western Tanzania

**DOI:** 10.1371/journal.pone.0086510

**Published:** 2014-01-29

**Authors:** Safari M. Kinung'hi, Pascal Magnussen, Godfrey M. Kaatano, Coleman Kishamawe, Birgitte J. Vennervald

**Affiliations:** 1 National Institute for Medical Research (NIMR), Mwanza Centre, Mwanza, Tanzania; 2 DBL-Centre for Health Research and Development, Department of Veterinary Disease Biology, University of Copenhagen, Copenhagen, Denmark; Queensland Institute of Medical Research, Australia

## Abstract

**Background:**

Malaria, schistosomiasis and soil transmitted helminth infections (STH) are important parasitic infections in Sub-Saharan Africa where a significant proportion of people are exposed to co-infections of more than one parasite. In Tanzania, these infections are a major public health problem particularly in school and pre-school children. The current study investigated malaria and helminth co-infections and anaemia in school and pre-school children in Magu district, Tanzania.

**Methodology:**

School and pre-school children were enrolled in a cross-sectional study. Stool samples were examined for *Schistosoma mansoni* and STH infections using Kato Katz technique. Urine samples were examined for *Schistosoma haematobium* using the urine filtration method. Blood samples were examined for malaria parasites and haemoglobin concentrations using the Giemsa stain and Haemoque methods, respectively.

**Principal Findings:**

Out of 1,546 children examined, 1,079 (69.8%) were infected with one or more parasites. Malaria-helminth co-infections were observed in 276 children (60% of all children with *P. falciparum* infection). Malaria parasites were significantly more prevalent in hookworm infected children than in hookworm free children (p = 0.046). However, this association was non-significant on multivariate logistic regression analysis (OR = 1.320, p = 0.064). Malaria parasite density decreased with increasing infection intensity of *S. mansoni* and with increasing number of co-infecting helminth species. Anaemia prevalence was 34.4% and was significantly associated with malaria infection, *S. haematobium* infection and with multiple parasite infections. Whereas *S. mansoni* infection was a significant predictor of malaria parasite density, P. *falciparum* and *S. haematobium* infections were significant predictors of anaemia.

**Conclusions/Significance:**

These findings suggest that multiple parasite infections are common in school and pre-school children in Magu district. Concurrent *P. falciparum*, *S. mansoni* and *S. haematobium* infections increase the risk of lower Hb levels and anaemia, which in turn calls for integrated disease control interventions. The associations between malaria and helminth infections detected in this study need further investigation.

## Introduction

Malaria, schistosomiasis and soil transmitted helminth infections (STH) are the most important parasitic infections in Sub-Saharan Africa, where a significant proportion of the populations including school children are exposed to these infections [1], [2], [3], [4]. They are particularly more prevalent in rural communities and are closely associated with poverty [5], [6], [7]. In Tanzania, these infections are a major public health problem among school and pre-school children [8], [9], [10]. Malaria, caused mainly by *P. falciparum* occurs throughout the country with varying levels of endemicity. Stable and perennial transmission occurs in the warm humid coastal regions and around the great Lakes [11], [12]. Schistosomiasis and STH infections also occur throughout Tanzania. Schistosomiasis is caused by *Schistosoma mansoni* and *Schistosoma haematobium* while the major STH infections are caused by the hookworms *Necator americanus* and *Ancylostoma duodenale*, *Ascaris lumbricoides* and *Trichuris trichiura* [8], [13], [14]. In the Lake Victoria basin, a prevalence of schistosomiasis exceeding 50% has been reported [8], [15], [16]. As a result of geographical overlap, malaria, schistosomiasis and the major STH (Hookworm, *Trichuris trichiura* and *Ascaris lumbricoides*) share not only the areas in which they occur, but also the human hosts [1], [4], [17], [18], [19], [20]. Children co-infected with these parasites develop less than optimal, have reduced learning and school achievements [21], [22] and have increased susceptibility to other infections [23], [24], [25]. Epidemiological studies have indicated that individuals co-infected with more than one parasite species are at risk of increased morbidity [26], [27], [28], [29] as well as at a risk of developing frequent and more severe disease due to interactions among the infecting parasite species [1], [23], [24], [30], [31]. Despite existence of contrasting evidence [32], there is increasing evidence suggesting that individuals infected by helminth infections are more likely to develop clinical *P. falciparum* malaria than helminth free individuals [23], [24], [33]. Concurrent parasitic infections also jointly contribute to anaemia. Hookworm and *T. trichiura* infections are associated with anaemia due to blood and iron loss into the intestinal tract while *S. mansoni* and *S. haematobium* infections cause blood loss in faeces and urine, respectively [34], [35]. Malaria contributes to decreased haemoglobin (Hb) concentrations and anaemia through a number of mechanisms including destruction of parasitized red blood cells, shortening of life span of non-parasitized red blood cells and decreased production of red blood cells in the bone marrow [36], [37]. Considering the limited number of studies on interactions between malaria and helminth co-infections in human populations, the present study was undertaken to investigate the epidemiology of malaria and helminth co-infections and the prevalence of anaemia in school and pre-school children in Magu district, North-western Tanzania.

## Methodology

### Study area and population

**Figure 1:**
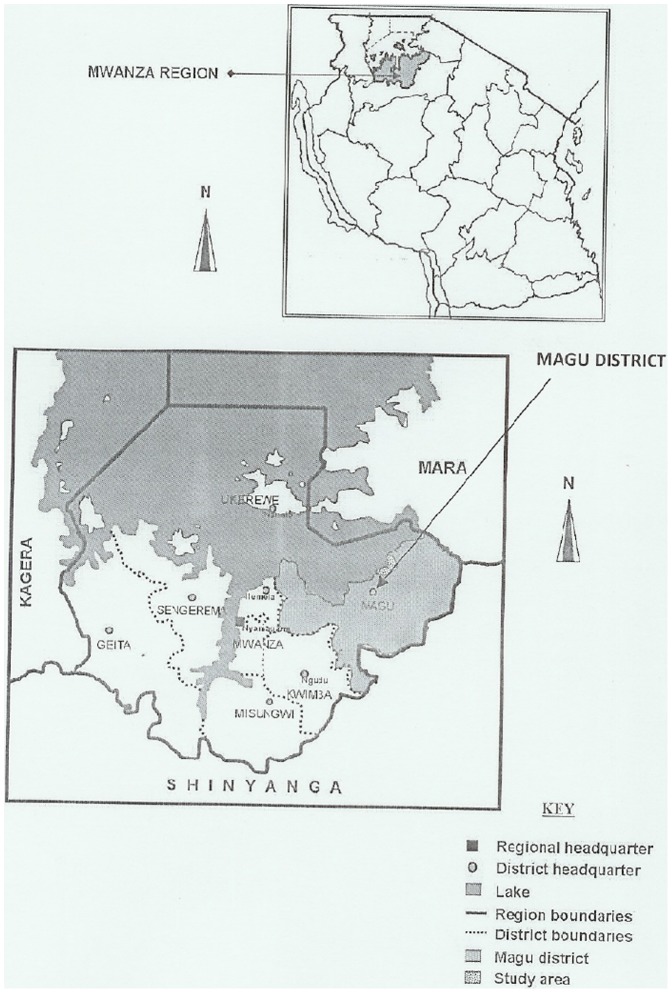
Administrative map of Tanzania showing the location of Mwanza region and the location of Magu district within Mwanza region.

The study was conducted in Magu district, North-Western Tanzania. Magu district lies between 2°10′ and 2°50′ South of Equator and 33° and 34° East of Greenwich. It has an area of 3075 km^2^ of which 1725 km^2^ (56.1%) is covered by Lake Victoria waters. Mean temperature ranges from 18°C to 20°C during the rainy season and 26°C to 30°C during the dry season. Rainfall is bimodal with the short rains between October to December and heavy rains between March and May. Mean annual rainfall ranges from 700 m to 1000 mm. In 2003, the district had a population of 416,113 people of whom 202,077 (48.6%) were males [38]. The predominant ethnic group in Magu district is the Wasukuma who practise subsistence farming (animal husbandry and crops) and fishing in Lake Victoria. According to hospital records, malaria remains the number one cause of hospital admissions and child morbidity and mortality in the district. Malaria transmission occurs throughout the year with peaks during the two rain seasons. Magu district has many water bodies particularly in areas lying in the Lake Victoria basin which are ideal for snail habitats and mosquito breeding. The district is hyper- to holoendemic for malaria with transmission occurring throughout the year. Schistosomiasis and soil-transmitted helminthiasis are also endemic in the district [8], [16]. The study took place between October to November, 2006. Six primary schools namely Mwamayombo, Nyashimo, Bulima, Milambi, Ihale and Ijitu were selected from the study area (Figure 1) and included in the study. From each selected school, school and pre-school children aged 3–13 years were selected and included in the study.

### Ethical statement

The study was approved by the Medical Research Coordination Committee (MRCC) of the National Institute for Medical Research (NIMR), Tanzania (Reference No. NIMR/HQ/R.8a/Vol. IX/355). Before commencement of the study, the research team conducted meetings with leaders, teachers and community members of all selected villages. During these meetings, the objectives of the study including the study procedures to be followed, samples to be taken, study benefits and potential risks and discomforts were explained. Informed consent for all children who participated in the study was sought from parents and legal guardians after they have been clearly informed about the study. Parental consent given from parents/guardians was written. Children were also requested to give assent and were informed of their right to refuse to participate in the study and to withdraw at any time during the study without jeopardizing their right of access to other health services. Invasive procedures such as collection of blood samples were fully explained to parents and children and were carried out using sterile disposable materials. All children found infected with any of the parasites *S. mansoni, S. haematobium*, soil-transmitted helminthiasis and *P. falciparum* and those found with ailments not targeted by the project were treated free of charge according to national guidelines. Study identification numbers were used instead of children names and information collected was kept confidential. Feedback to the study population in the form of dissemination workshops was conducted during the course of the study.

### Collection and examination of stool, urine and blood samples

Children were provided with plastic containers and requested to bring stool and urine samples on two consecutive days at about 10:00am in the morning. Stool samples were examined for *S. mansoni* and intestinal helminths (*T. trichiura, A. lumbricoides* and hookworm) using the Kato Katz method [39]. Duplicate smears (41.7 mg) were prepared from each stool sample. Intensity of infection for *S. mansoni* and intestinal helminths were expressed as the mean eggs per gram of faeces (epg) of the two samples (four smears). Urine samples were examined for *S. haematobium* eggs in 10 ml of urine according to the nucleopore filtration method [40]. Blood samples (approximately 3 ml) were collected using plain vacutainer tubes or disposable syringes. Thick blood smears were prepared, stained with Giemsa and examined microscopically for malaria parasites. Haemoglobin concentrations (Hb) were determined using a portable HaemoCue photometer. Anaemia was defined as Hb<120 g/L and Hb<80 g/L as severe anaemia. Quality control was performed by re-examining 10% randomly selected blood slides, urine filters and Kato smears by an experienced independent technician.

### Data analysis

Data were double entered into Dbase V software (Borland International, Scotts Valley, California, USA) and analyzed using STATA Version 10 (STATA Corp., Texas, USA). Parasite counts were normalized by log transformation, averaged and then back transformed to the original scale. Infection intensities were calculated as geometric mean of eggs per gram of faeces for *S. mansoni* and hookworm infections, eggs per 10 ml of urine for *S. haematobium* and parasites per microlitre of blood for *P. falciparum* based on positive samples only. The student's t-test and one way analysis of variance (ANOVA) was used to compare geometric mean parasite counts and mean Hb concentrations where two or more than two groups were compared, respectively. For parasite counts, the student's t-test and ANOVA were performed on log transformed data of positive samples only whereas for Hb concentrations the students t-test and ANOVA were performed for all samples examined on original scale. The Chi-square test was used to compare proportions and to test for association between malaria prevalence, anaemia prevalence and prevalence of helminth infections between exposure groups. In the multivariate analysis, presence or absence of infection or anaemia was compared among schools, age groups, sexes and other infections using logistic regression analysis fitted as a generalized linear model with a logit link function and adjusting for possible clustering among siblings. All predictors were initially tested for significance separately and then jointly in a multi-variable model. Except for box plots which were drawn using STATA version 10, all other graphs were drawn using MS-Excel software. Tests were considered statistically significant at p<0.05.

## Results

A total of 1615 school and pre-school children were examined. Pre-school children were 372 or 23% of all children examined. Children where complete information was available were included in the analysis (1546) of whom 759 (49.1%) were boys. Overall mean age was 7 years.

### Parasite prevalence and infection intensities

Out of the 1546 children included in the analysis, 1079 (69.8%) were infected with at least one of the parasites *P. falciparum, S. mansoni, S. haematobium*, hookworm and *T. trichiura. S. mansoni* infections were generally light to moderate with only 59 children (9.6%) being heavily infected (epg≥400). Whereas 94 children (3.8%) had heavy *S. haematobium* infections (≥50eggs/10 ml of urine), all hookworm infections were light (epg<2000). Three children (0.2%) were infected with *T. Trichiura* and *Ascaris lumbricoides* infections were absent. *S. mansoni* infection was the most prevalent parasite followed by *P. falciparum* and *S. haematobium*. Hookworm was the least prevalent parasite. The prevalence of individual parasite species and the respective infection intensities are shown in table 1.

**Table 1:** Overall prevalence (n = 1546) and infection intensities (expressed as geometric mean parasite count of positive samples) of parasitic infections in school and pre-school children in Magu district, Tanzania.

	Malaria	*S. mansoni*	*S. haematobium*	Hook worm
No. infected	460	613	305	245
Prevalence (%)	29.8	39.7	19.7	15.8
Geometric mean parasite count	626.6 (547.1–717.5)	50.7 (45.1–57.0)	16.1 (13.2–19.7)	53.7 (45.6–63.3)
Maximum density	29840	3513	642	1265

The prevalence of *S. mansoni, S. haematobium* and hookworm infections differed significantly across age groups (p<0.001) whereby older children (6–8 years and 9–13 years) had higher prevalence of infection compared to younger children (3–5 years). Likewise, the infection intensity of *S. mansoni* and hookworm differed significantly across age groups (p<0.001) whereby children in higher age groups had higher parasite loads compared to children in the lower age group. The prevalence and infection intensity of *S. mansoni, S. haematobium* and hookworm also differed significantly across schools (p>0.001). Malaria prevalence varied considerably among schools (p<0.001) being highest in Mwamayombo and Milambi compared to other schools. Younger children had higher malaria parasite density compared older children (p<0.001).

### Prevalence of co-infections

**Figure 2:**
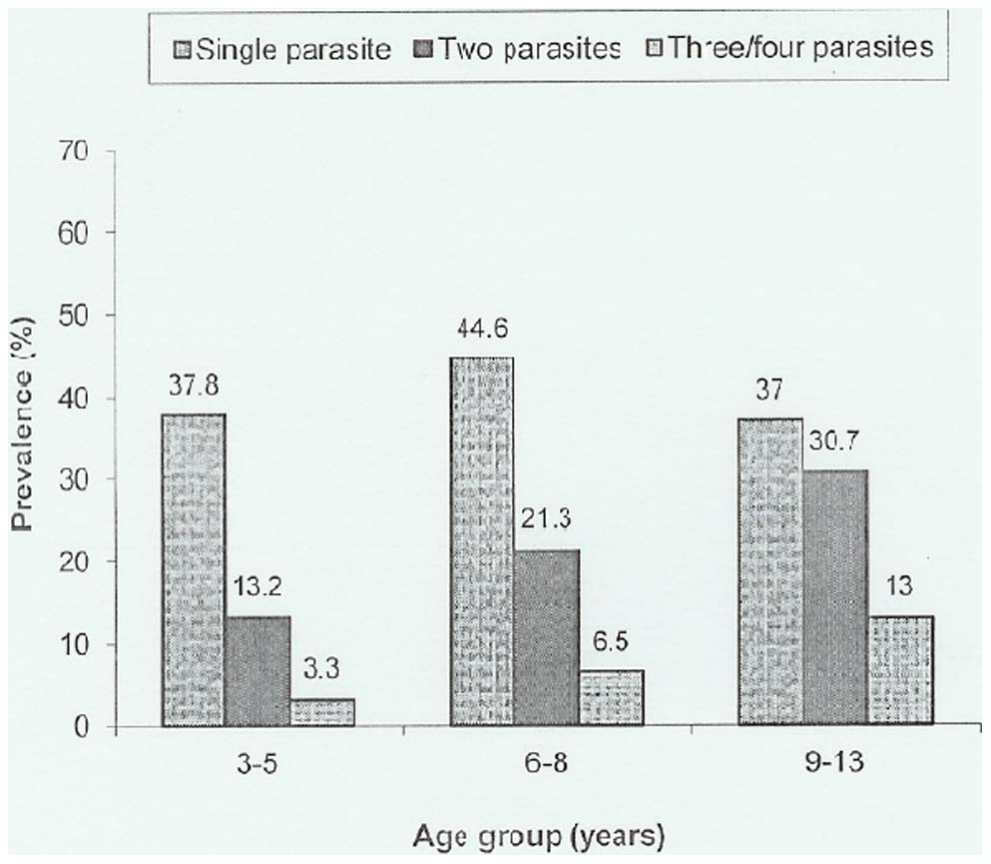
Prevalence of single and multiple parasitic infections by age groups (n = 1079).

Out of the 1079 infected children, 430 (39.9%) harboured more than one parasite species. Overall, *S. mansoni* infections occurred as single as well as a multiple species infection in almost equal proportions (18.8% and 20.95, respectively). *P. falciparum, S. haematobium* and hookworms infections occurred more frequently as multiple species infections than single species infections. Figure 2 summarize the prevalence of single and multiple parasite species infections by age groups.

Figure 2 shows that multiple parasite infections occurred more frequently in older children (9–13 years) compared to younger children (3–5 and 6–8 years) (χ^2^ = 51.07, p<0.001).

### Associations between parasite infections

The prevalence of helminth co-infections among *P. falciparum* infected children was 60% (276/460). The most common parasite combinations were *P. falciparum* and *S. mansoni* (27.2%), *P. falciparum* and *S. haematobium* (10.2%), *P. falciparum* and hookworm (7.4%), *P. falciparum*, *S. mansoni* and *S. haematobium* (7%), *P.falciparum, S.mansoni* and hookworm (6.5%) and *P. falciparum*, *S. haematobium* and hookworm (3.0%). Malaria and helminth co-infections occurred more frequently in older children (9–13 years) compared to younger children (3–5 and 6–8 years) and the difference was significant (χ^2^ = 19.34, p<0.001). Malaria parasites were significantly more prevalent in hookworm infected children than in hookworm free children (35.1% vs 28.8%) (χ^2^ = 3.98, p = 0.046). However, this association turned to be non-significant when multivariate logistic regression analysis was performed while adjusting for other confounding factors (OR = 1.320, p = 0.064). Children with hookworm infection were more likely to be infected with *S. haematobium* (χ^2^ = 7.52, p<0.01) compared to children who were not infected with hookworm. Further, children infected with hookworm were also likely to be infected with *S. mansoni* (χ^2^ = 6.40, p = 0.011) compared to children who were not infected with hookworm. The prevalence of malaria parasites tended to increase with increasing number of co-infecting helminth species. The prevalence of malaria parasites was 29%, 35% and 41.2% in children harbouring one, two and three helminth specie, respectively, compared to 28.3% in helminth free children. However, the difference was not significant (χ^2^ = 5.63, p = 0.131).

### Association between malaria parasite density and helminth infections

**Figure 3:**
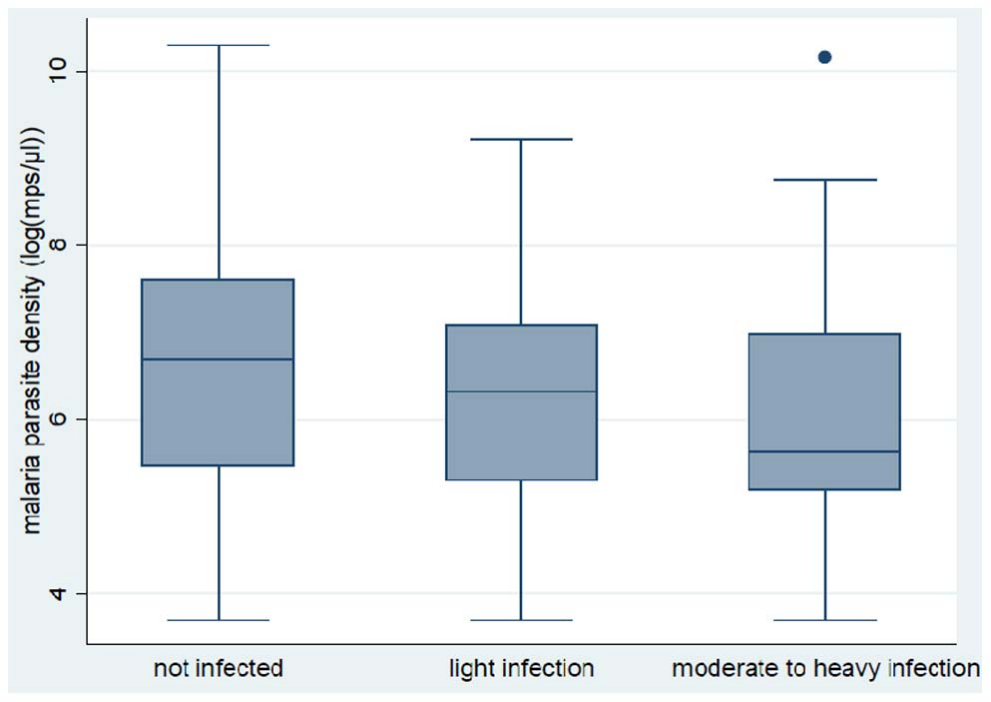
Box and whisker plot showing the relationship between median and range of log transformed values of malaria parasite density (Log(mps/µL)) and *S. mansoni* infection status (categorised as not infected, light infection and moderate to heavy infection) (N = 1546). The thick line inside each box represents the median value. The lower and upper edge of each box indicates the 25^th^ and 75^th^ percentiles, respectively. The lower and upper whiskers represent the lower and upper values (range), respectively, excluding outliers.

**Figure 4:**
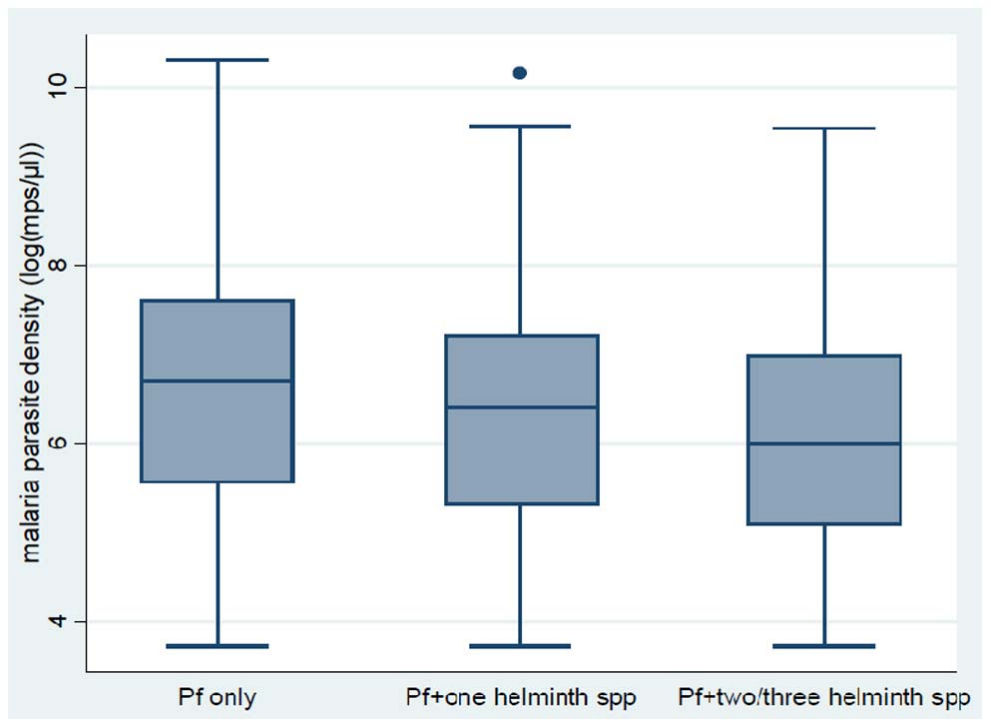
Box and whisker plot showing the relationship between median and range of log transformed values of malaria parasite density (Log(mps/µL)) and helminth infection status (N = 1546). Pf only = *P. falciparum only*; Pf+one helminth spp = *P. falciparum* plus onehelminth specie; Pf+two/three helminth spp = *P. falciparum* plus two or three helminth species.

Except for hookworm infection, malaria parasite density was negatively correlated with helminth infections (prevalence and infection intensity). Figure 3 shows the relationship between malaria parasite density and *S. mansoni* infection while figure 4 shows the relationship between malaria parasite density and the number of co-infecting helminth species.

Malaria parasite densities tended to decrease with increasing infection intensity of *S. mansoni*. Geometric mean malaria parasite density for children without *S. mansoni* infection was 745 (95% CI 633–879) and was significantly higher compared to 551 (95% CI 434–700) and 399 (95% CI 297–534) for children with light and moderate to heavy *S. mansoni* infection, respectively (F = 6.9, p<0.01) (Fig. 3). Figure 4 shows the relationship between malaria parasite densities and overall helminth infections. Malaria parasite densities tended to decrease with increasing number of co-infecting helminth species. Geometric mean malaria parasite density for children without any helminth infection (malaria only) (n = 184) was 779 (95% CI 640–948), significantly higher compared to 604 (95% CI 499–731) and 425 (95% CI 319–566) for children with one and two or more co-infecting helminth species, respectively (F = 4.0, p<0.01). On the other hand, children infected with hookworm had higher infection intensity of *S. mansoni* compared to children without hookworm infection (t = −3.19, p<0.01).

### Prevalence of anaemia and association with infection status

**Figure 5:**
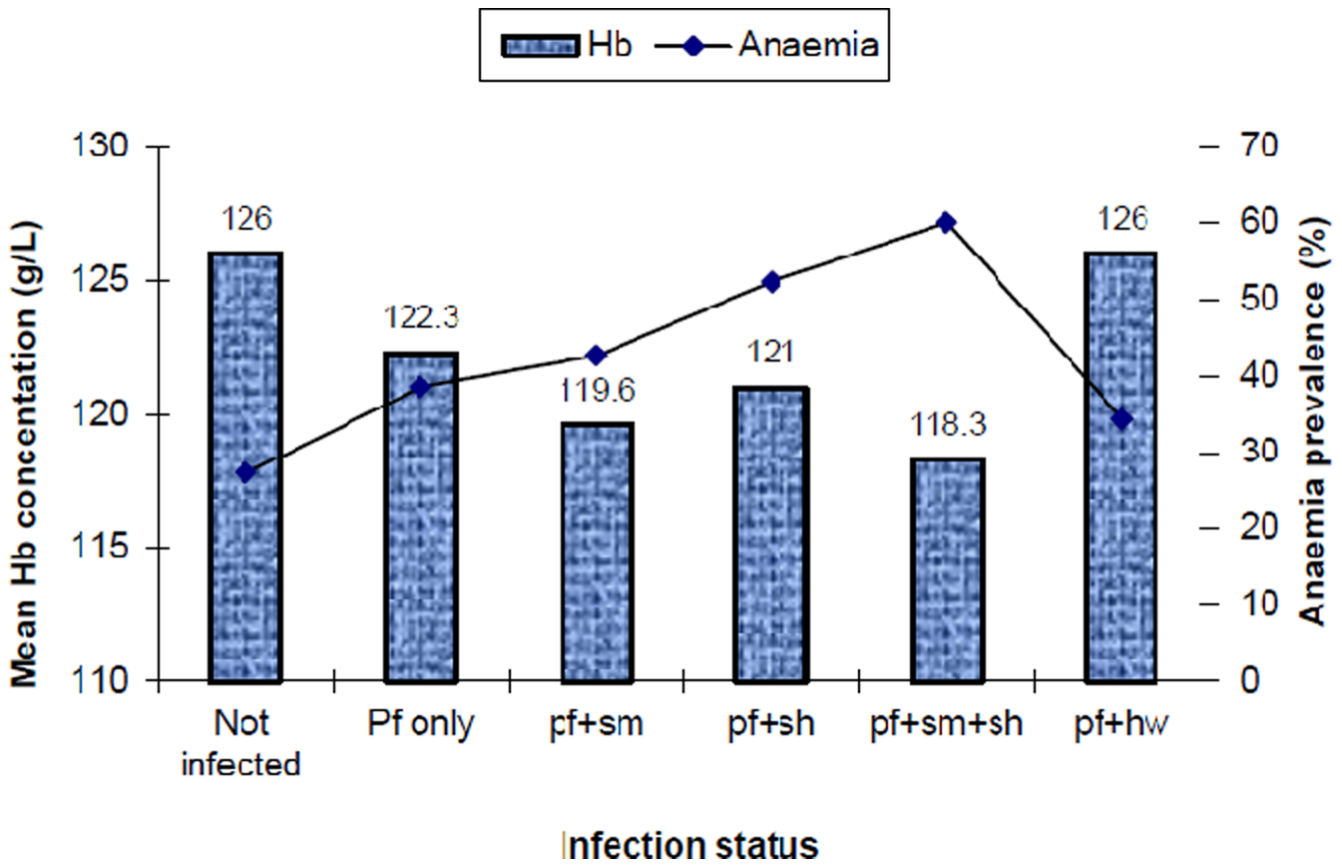
Prevalence of anaemia and mean haemoglobin levels in relation to malaria and helminth co-infections (n = 1546). Infection status: Pf = *P. falciparum*; *Sm = S. mansoni*; Sh = *S. haematobium*; Hw = Hookworms.

Out of the 1546 children, 532 (34.4%) were anaemic and only 16 (1.0%) were severely anaemic. Overall mean Hb concentration was 123.7 (95% CI 123.0–124.4). The prevalence of anaemia was significantly associated with malaria infection (χ^2^ = 15.58, p<0.001) and *S. haematobium* infection (χ^2^ = 16.34, p<0.001). For *P. falciparum* and *S. haematobium* infections, mean Hb levels decreased significantly with increasing infection intensities (p<0.01). Children who were not infected with any parasite had the highest mean Hb levels (126.0, 95% CI 124.7–127.2) and hence the lowest prevalence of anaemia (27.2%). Except for *P. falciparum* and *hookworm* co-infections, children who were infected with more than one parasite species tended to have lower mean haemoglobin levels and hence higher prevalence of anaemia compared to children infected with one parasite. The highest prevalence of anaemia (60%) was observed in children co-infected with three parasites *P. falciparum*, *S. mansoni* and *S. haematobium* (Figure 5). There was a significant difference in the prevalence of anaemia and mean haemoglobin levels between uninfected children and those infected with one or more parasites (p<0.01).

### Predictors of anaemia

Multivariate logistic regression analysis was performed to identify predictors of anaemia. Variables included in the analysis were age group, sex, malaria infection, *S. mansoni* infection, *S. haematobium* infection, hookworm infection and the presence of any helminth infection. The results of the final model are summarised in table 2.

**Table 2:** Results of multivariate logistic regression analysis showing important predictors of anaemia with adjusted odds ratios and p-values (N = 1546).

Independent variable	Categories	Adjusted OR (95% CI)	P-Value
Age group	3–5 years	1	
	6–8 years	0.996 (0.763–1.300)	0.974
	9–13 years	0.930 (0.649–1.320)	0.688
Sex	Male	1	
	Female	0.860 (0.695–1.064)	0.166
Malaria infection	Not infected	1	
	Light infection	1.502 (1.188–1.899)	<0.01
	Heavy infection	2.241 (1.154–4.352)	0.017
*S. haematobium* infection	Not infected	1	
	Light infection	1.533 (1.133–2.074)	<0.01
	Heavy infection	2.01 (1.312–3.075)	<0.01

*P. falciparum* and *S. haematobium* infections were significant predictors of anaemia after adjusting for age and sex (Table 2).

## Discussion

Malaria, schistosomiasis and STH are a major public health problem particularly to school and pre-school children in Sub-Saharan Africa where their occurrence as multiple species infections is known to be the norm. Understanding the epidemiology of these infections among school and pre-school children and their joint contribution to lower haemoglobin levels and anaemia is important as findings may support design of integrated disease control strategies. Results of this study demonstrated that malaria, schistosomiasis and soil-transmitted helminth infections are prevalent in school and pre-school children in Magu district and co-infections of these parasites were common. These findings are supported by other studies in the Sub-Saharan Africa [41], [42], [43], [44], [45]. The most prevalent parasite species in the studied population were *S. mansoni*, *P. falciparum* and *S. haematobium*. The major STH infections hookworm and *T. trichiura* were the least prevalent. *Ascaris lumbricoides* was not detected in the current study. This observation concurs with findings of the study of Lwambo *et al* [8] which reported this specie to be rare and is in line with the known distribution of *A. lumbricoides* in Sub-Saharan Africa [46]. The observed prevalence of *S. mansoni* and *S. haematobium* are in accordance with previous studies in the area and is related to the occurrence of the snail intermediate hosts for *S. mansoni* and *S. haematobium* and their ecological preferences [8], [14], [15], [47]. The low prevalence of STH infections in the studied population could be as a result of the relatively younger age of most of children examined as the prevalence of STH particularly hookworm peaks in early adulthood (34). For schistosomiasis and hookworm infections, the observed prevalence and infection intensity were generally age dependent which reflects the fact that infection levels are explained by water contact patterns, duration of exposure to infection and acquired immunity [35], [48], [49], [50]. The study also observed significant variation among schools of both prevalence and infection intensities of *S. mansoni* and *S. heamatobium* which could be explained by variations in exposure, focal nature of schistosomiasis and the over-dispersed distribution of heavy and light infections between and within communities [35]. Malaria parasite densities decreased with increasing age which is a normal trend in malaria endemic areas and is related to development of anti-malarial specific immunity [51].

In addition to single parasite infections, this study also demonstrated that co-infections are very common in the study area and interactions exist among them. Majority of children who were infected with *P. falciparum* were concurrently infected with one or more helminth species. In the bivariate analysis, hookworm infection was found to have a positive association with malaria infection and malaria parasite density. However, this association was not confirmed by multivariate logistic regression analysis and hence needs further investigation. Previous studies which found of a positive association between malaria and hookworm infection include Humphries *et al* [18], Nacher *et al* [23], Hillier *et al* [25], Spiegel *et al* [33], and Yatich *et al* [52]. However, the study of Shapiro *et al* had contrasting findings [32]. Studies which favor the existence of a positive association between hookworm and malaria infection propose various underlying mechanisms. There is evidence suggesting that environmental, socio-economic and behavioural factors could act as shared risk factors for exposure to both infections [4], [19], [25], and the involvement of immunological mechanisms which may lead to increased susceptibility of helminth infected individuals to *P. falciparum* infection [24], [33], [53]. On the other hand, this study showed a negative association between *S. mansoni* and *S. haematobium* infections and malaria parasite intensity, in line with observations made by Lyke *at al* [54] and Briand *et al* [49] in Mali and Senegal, respectively. The study of Lyke *et al* [54] demonstrated that *S. haematobium* infected children had lower geometric mean malaria parasite density compared to children without *S. haematobium* infection. The study of Briand *et al* [49] showed that children with light infection of *S. haematobium* had lower *P. falciparum* parasite densities compared to those not infected. One possible explanation for this observation could be cross reactivity between anti-*P. falciparum* antibodies and anti-schostosomal antibodies as has been reported for *S. mansoni* and *P. falciparum* specific antibodies [55], [56], [57].

Anaemia was prevalent in the study area though at a relatively low level compared to what was reported by the study of Lwambo *et al* [8] which reported an overall prevalence of anaemia of up to 62.4%. This observation may reflect a changing pattern in prevalence of anaemia and the distribution of helminth infections (prevalence and infection intensity) in the study area. Another possible explanation could be the difference in age distribution of children who participated in the two studies. While the current study enrolled children between 3 to 13 years, the study of Lwambo *et al* [8] enrolled children between 7 to 20 years. Majority of anaemia cases in the current study were moderate. Only 16 children (1%) had severe anaemia probably due to the fact that majority of helminth infections were also light. This observation is in agreement with findings of Ajanga *et al* [15], Lwambo *et al* [58] and Koukounari *et al* [59], who observed that anaemia due to heminth infections is dependent on intensity of infection. As expected and in accordance with findings of other studies [8], [34], [48], [60], [61], [62], [63], lower Hb concentrations and anaemia was associated with single and multiple parasitic infections. Although the aetiology of anaemia is multifactorial, parasitic infections are known to be among major causes [41], [59], [60]. While *P. falciparum* infection causes anaemia through complex mechanisms including destruction of parasitized red blood cells, decreased production of red blood cells (RBCs) and/or dyserythropoiesis [36], [51], [64], *S. mansoni, S. haematobium* and hookworm infections cause anaemia through chronic blood loss [34], [35], [41], [48]. In contrast to previous studies [7], [8], [48], [60], [62], [65], [66], hookworm was found not to be associated with anaemia probably due to the relatively low infection intensities of hookworm infection detected in the studied population. Further, multiple logistic regression analysis showed that malaria and *S. haematobium* infections were predictors of anaemia, a finding which indicates that in addition to the known effect of single parasite species on anaemia, multiple parasite infections can interact to enhance the risk of anaemia. Interestingly, the highest prevalence of anaemia (60%) was observed in children concurrently infected with *P. falciparum*, *S. mansoni* and *S. haematobium*, and in children concurrently infected with *P. falciparum* and *S. haematobium* (52.3%). Anaemia was also more prevalent in children concurrently infected with three or four parasites compared to those with only one or no parasite infection. These observations demonstrate a possible synergistic interaction of *P. falciparum, S. mansoni* and *S. haematobium* and multiple parasite infections as the aetiology of anaemia. Limitations of the current study in elucidating associations between malaria and helminth co-infections include the lack of information on household, socio-economic status and environmental factors which have been shown to influence occurrence of co-infections by other studies [19], [52]. The lack of information on other causes of anaemia such as malnutrition was another limitation.

Overall, results of this study have demonstrated that malaria, schistosomiasis and STH infections are prevalent in school and pre-school children in Magu district and that polyparasitism is also very common. These findings also suggest that concurrent *P. falciparum*, *S. mansoni* and *S. haematobium* infections increase the risk of lower Hb levels and anaemia which in turn calls for integrated disease control interventions. The associations between malaria and helminth infections detected in this study were not conclusive and hence needs further investigation.
